# A novel group of diverse Polinton-like viruses discovered by metagenome analysis

**DOI:** 10.1186/s12915-015-0207-4

**Published:** 2015-11-11

**Authors:** Natalya Yutin, Sofiya Shevchenko, Vladimir Kapitonov, Mart Krupovic, Eugene V. Koonin

**Affiliations:** National Center for Biotechnology Information, National Library of Medicine, National Institutes of Health, Bethesda, MD 20894 USA; Unité Biologie Moléculaire du Gène chez les Extrêmophiles, Department of Microbiology, Institut Pasteur, Paris, France

## Abstract

**Background:**

The rapidly growing metagenomic databases provide increasing opportunities for computational discovery of new groups of organisms. Identification of new viruses is particularly straightforward given the comparatively small size of viral genomes, although fast evolution of viruses complicates the analysis of novel sequences. Here we report the metagenomic discovery of a distinct group of diverse viruses that are distantly related to the eukaryotic virus-like transposons of the Polinton superfamily.

**Results:**

The sequence of the putative major capsid protein (MCP) of the unusual linear virophage associated with *Phaeocystis globosa* virus (PgVV) was used as a bait to identify potential related viruses in metagenomic databases. Assembly of the contigs encoding the PgVV MCP homologs followed by comprehensive sequence analysis of the proteins encoded in these contigs resulted in the identification of a large group of Polinton-like viruses (PLV) that resemble Polintons (polintoviruses) and virophages in genome size, and share with them a conserved minimal morphogenetic module that consists of major and minor capsid proteins and the packaging ATPase. With a single exception, the PLV lack the retrovirus-type integrase that is encoded in the genomes of all Polintons and the Mavirus group of virophages. However, some PLV encode a newly identified tyrosine recombinase-integrase that is common in bacteria and bacteriophages and is also found in the Organic Lake virophage group. Although several PLV genomes and individual genes are integrated into algal genomes, it appears likely that most of the PLV are viruses. Given the absence of protease and retrovirus-type integrase, the PLV could resemble the ancestral polintoviruses that evolved from bacterial tectiviruses. Apart from the conserved minimal morphogenetic module, the PLV widely differ in their genome complements but share a gene network with Polintons and virophages, suggestive of multiple gene exchanges within a shared gene pool.

**Conclusions:**

The discovery of PLV substantially expands the emerging class of eukaryotic viruses and transposons that also includes Polintons and virophages. This class of selfish elements is extremely widespread and might have been a hotbed of eukaryotic virus, transposon and plasmid evolution. New families of these elements are expected to be discovered.

**Electronic supplementary material:**

The online version of this article (doi:10.1186/s12915-015-0207-4) contains supplementary material, which is available to authorized users.

## Background

Metagenomic sequences are a treasure trove of novel genes and genomes [1–5]. At present, after the release of the massive data from the Global Ocean Survey [6] and especially the more recent Tara project [7], the amount of metagenomic sequences already substantially exceeds the size of the regular sequence databases such as GenBank. Perhaps even more importantly, metagenomic data sets have not only a quantitative but also a qualitative advantage over genomic databases. Metagenomes are not constrained by the inherent requirement of traditional sequence databases that for genome sequencing, an organism has to be clearly identified and, at least in the case of microbes, grown in the laboratory. Metagenomes, at least in principle, are unbiased representations of the environs from which they originate (apart from possible sequencing biases). Herein, also lies the intrinsic weakness of metagenomics: technically, it is never known which organism a given sequence comes from. Sequences with high similarity to those from known organisms are readily assignable but those from truly novel genomes can be difficult to place. A further difficulty for metagenomic discovery is the correct assembly of long contigs. Complete assembly of a typical bacterial or archaeal genome of several megabases is usually impractical, even when extremely deep sequencing is performed, and assignment of multiple contigs to a single genome is a separate, non-trivial problem.

The difficulties of metagenomic genome discovery are alleviated to a considerable extent when it comes to discovery of new virus genomes [8–10]. Viral genomes are relatively small, for many important and abundant virus families, only several kilobases (kb) long, so that assembly of complete viral genome is often feasible. Furthermore, viruses possess signature genes, such as those encoding capsid protein, that provide for assignment of novel genomes to a particular family of viruses even in the absence of close relatives. The flip side of the coin is that viruses typically evolve much faster than cellular life forms so that finding homologs of virus genes is often challenging. Hence the special importance of advanced methods for sequence and structure analysis in virus discovery.

Virus metagenomics is a young research direction but can already boast several stories of conspicuous success in the discovery of novel groups of (putative) viruses with unexpected features. Among the most notable ones is the identification of the class of chimeric single-stranded (ss) DNA viruses that combine genes from known families of positive-strand RNA viruses and ssDNA viruses. The first discovery of a chimeric virus [11] was followed by a systematic effort on metagenome mining, which led to the identification of several diverse groups of naturally chimeric virus genomes [12], and finally, by a serendipitous discovery of an actual virus with a chimeric genome [13]. These findings have substantially enriched the available collection of ssDNA virus genomes, but more importantly, have changed the existing picture of the evolution of this class of viruses. An equally spectacular discovery of metagenomics is the identification of a novel bacteriophage that is more abundant than any other phage in the human gut but has remained unnoticed until metagenome mining has become highly efficient [14]. Yet another notable case in point is the use of a signature enzyme of the giant viruses in the family *Mimiviridae*, glutamine-hydrolyzing asparagine synthase, as the bait to search metagenomic databases, resulting in a substantial expansion of this virus family [15]. There are more examples of successful application of metagenome mining for the discovery of new viruses but the above should suffice to illustrate the utility and promise of this approach. Although virus metagenomics cannot provide direct data on the structure and functionality of the discovered viruses, through genome analysis, it yields a wealth of predictions that are amenable to experimental validation.

We are interested in a class of viruses and virus-like transposable elements that includes Polintons (Mavericks) and virophages. Polintons are large (genomes of 15 to 20 kb, the largest among the eukaryotic transposons), self-replicating transposable elements that are integrated into the genomes of diverse unicellular and multicellular eukaryotes in highly variable numbers of copies [16–20]. All Polintons encode a protein-primed DNA polymerase and a retrovirus-like integrase (hence the name of these elements: POLINTons). The majority of Polintons also encode a homolog of the DNA-packaging ATPase and maturation protease that are characteristic of diverse double-stranded (ds) DNA viruses. Thus, Polintons have been often considered virus-like transposons although no structural proteins have been initially detected. To resolve this conundrum, we have recently performed an exhaustive computational analysis of the Polinton-encoded protein sequences and have shown that most of the Polintons encode putative major and minor capsid proteins (MCP and mCP, respectively) [21]. Although sequence similarity between these proteins and capsid proteins of other viruses, such as the large and giant viruses that constitute the putative order “Megavirales”, is low, homology modeling indicates that MCP and mCP respectively adopt intact double and single jelly-roll folds, strongly suggesting that Polintons are capable of forming virions [21]. Thus, we have hypothesized that Polintons lead a dual life style and, in addition to behaving like typical transposons, are capable of forming virions, hence the proposed name polintoviruses [21, 22]. However, the actual polintovirus virions remain to be identified and therefore below we stick to the term Polintons unless putative virus particles are explicitly considered.

A distinct Polinton-like transposable element called Tlr1 is present in the genome of the ciliate *Tetrahymena thermophila* [23]. Although Tlr1 does not encode the DNA polymerase, it shares with *bona fide* Polintons the retroviral-type integrase (RVE), DNA-packaging ATPase and both capsid proteins [21]. In addition, Tlr1 encodes several other proteins, including a PIF1-like superfamily 1 helicase, that are shared with some members of the proposed order “Megavirales” [22, 23].

Extensive comparative analysis of the genomes of dsDNA viruses infecting eukaryotes as well as dsDNA plasmids and transposons has led to an evolutionary scenario in which polintoviruses evolved directly from bacteriophages of the family *Tectiviridae* and played a central role in the origin and evolution of diverse selfish elements in eukaryotes including giant viruses of the proposed order “Megavirales” [22, 24].

The group of viruses that arguably includes the closest relatives of the Polintons are the virophages, unusual small DNA viruses that parasitize on giant viruses of the family *Mimiviridae* [25–27]. The genome size and organization of the virophages show striking similarity to the Polintons (polintoviruses) except that the virophage genomes are apparently circular [28, 29]. The virophage genomes are about 20 to 30 kb in size and encode the MCP and mCP, the packaging ATPase and the maturation protease along with a heterogeneous set of other genes [29]. Mavirus-like virophages also encompass genes for a protein-primed DNA polymerase (pDNAP) and a RVE integrase [28]. Thus, the Maviruses effectively qualify as polintoviruses except that so far integration into the host genome has not been demonstrated.

Originally, virophages have been isolated as virus particles from preparations of giant viruses. Efforts on discovery of new virophages in metagenomic databases have yielded several relatives of the previously characterized virophages, primarily from the thermal Yellowstone Lake [30, 31]. In addition, our previous search of the metagenomic sequences available in GenBank has led to the identification of a putative novel group of virophages in the sheep rumen metagenome [32]. Notably, these rumen virophages (RVP), in addition to the typical virophage major (but not minor) capsid protein, ATPase and protease, encode a Polinton-type pDNAP and thus appear to be hybrids between virophages and Polintons.

So far all searches for putative new virophages in metagenomic sequences have employed the virophage MCP as the initial bait [30–32]. The virophage MCP is a structurally highly derived version of the double jelly-roll fold, to the extent that sequence similarity with other capsid proteins, including those from Polintons, is virtually undetectable [33]. Therefore, these searches appear to be inherently limited with respect to the range of viruses that can be detected, i.e. are likely to identify only viruses that possess the virophage variety of MCP.

An unusual putative virophage has been discovered in DNA preparations of *Phaeocystis globosa* virus (PGV) infecting an abundant marine haptophyte (chromist alga) [34]. The PGV virophage (PgVV) has a linear genome of approximately 20 kb containing long terminal inverted repeats (TIR) and apparently can integrate into the PGV genome. Only three PgVV genes have been reported to encode proteins sharing significant sequence similarity to virophage proteins, namely a predicted primase, an endonuclease and an uncharacterized protein [34]. However, in the process of searching for potential capsid proteins of Polintons, we identified a candidate MCP of PgVV, a distant member of the polintovirus MCP family [21]. Given the distinct features of PgVV that differentiate it from both the characterized virophages and Polintons, we performed exhaustive searches of genomic and metagenomic sequences using the putative PgVV MCP as a bait. These searches, followed by extensive analysis of the retrieved contigs, yielded a diverse group of putative novel viruses.

## Results

### Sequence database screening for putative PgVV-like virus genomes

**Fig. 1:**
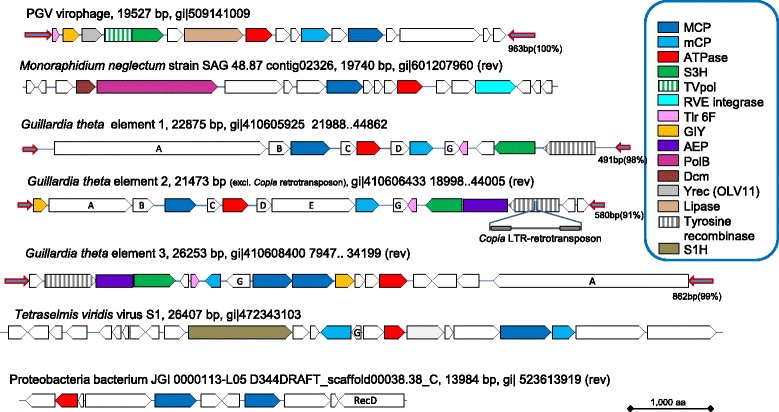
Genome architectures of the Polinton-like viruses (PLV): complete genomes from identified sources. Genes are shown roughly to scale. Homologous genes are color-coded as shown in the inset. Homologous genes without predictable function (activity) are marked by same letters. Arrows represent terminal inverted repeats; their lengths and percent identity are indicated near the rightmost repeat. AEP, archaeo-eukaryotic primase; Dcm, methyltransferase of the Dcm family; GIY, GIY-YIG family nuclease; MCP, major capsid protein; mCP, minor capsid protein; PolB, protein-primed polymerase of family B; primpol, primase-polymerase; S1H, superfamily 1 helicase (distinct from the Tlr helicase in Figs. 2 and 3); S3H, superfamily 3 helicase; TVpol, transposon-viral polymerase; Yrec (OLV11), OLV11-like tyrosine recombinase (integrated PLV from *G. theta* encode a distinct tyrosine recombinase only distantly related to the OLV11-like family)

The following strategy was developed for comprehensive identification of the putative viruses encoding a PgVV-like putative MCP in genomic and metagenomic databases (see Methods for details). The sequence of the predicted MCP of PgVV was first used as a query in a PSI-BLAST search of the non-redundant protein sequence (nr) database at the NCBI. This search detected homologs of the PgVV MCP in five eukaryotic genomes: the algae *Monoraphidium neglectum* (gi|761972244); *Aureococcus anophagefferens* (gi|676398223); *Chlamydomonas reinhardtii* (gi|159489398); and *Guillardia theta* CCMP2712 (gi|551629560, gi|551640847, gi|551645334) (three homologs in the latter genome); and the carnivorous plant *Genlisea aurea* (gi|527182119). Examination of the genomic surroundings of the putative MCP genes resulted in the identification of several genes typical of Polintons in *M. neglectum* and *G. theta* (Fig. 1; see details below), whereas the other three genomes contained solitary MCP genes, conceivably remnants of genome invasion by PgVV-related elements. All PgVV-like MCP proteins were used as queries for TBLASTN searches against two metagenomic databases, CAMERA and marine metagenomes. All hits were collected, translated (the camera hits were assembled before translation), and the resulting protein sequences were employed as queries to search the nr database using BLASTP. Those metagenomic sequences, for which the encoded protein produced the best hits to one of the query MCP proteins, were retained. The collected metagenomic sequences were extended whenever possible using alternating cycles of BLASTN searches and assembly with Geneious (see Methods for details). This procedure allowed us to extend even some of the Tara Oceans sequences that have been assembled prior to database submission.

The sequences of the PgVV MCP homologs that were collected through the iterated procedure described above were used as queries in an exhaustive search for homologous MCP in metagenomes. Specifically, 20 diverse representatives were chosen using BLASTClust; for each of these, the TBLASTN search (e-value ≤10) against marine metagenomes was repeated. All contigs with hits were translated and subjected to two searches, namely BLASTP against nr and a profile-based version of TBLASTN against the marine metagenomes, with the position-specific scoring matrix derived from the alignment of all detected MCPs used as the query. The contigs encoding proteins with best hits to one of the identified MCPs or matching the MCP profile, were collected. This procedure yielded nearly 300 marine metagenome contigs encoding putative PgVV-like MCP. The 20 longer contigs that contained additional genes homologous to genes of polintoviruses or virophages were examined in detail (see Additional file 1), primarily by exhaustive analysis of the encoded protein sequences using sensitive database search methods such as PSI-BLAST and HHpred.

### Genomic architectures and gene complements of putative novel Polinton-like viruses

**Fig. 2:**
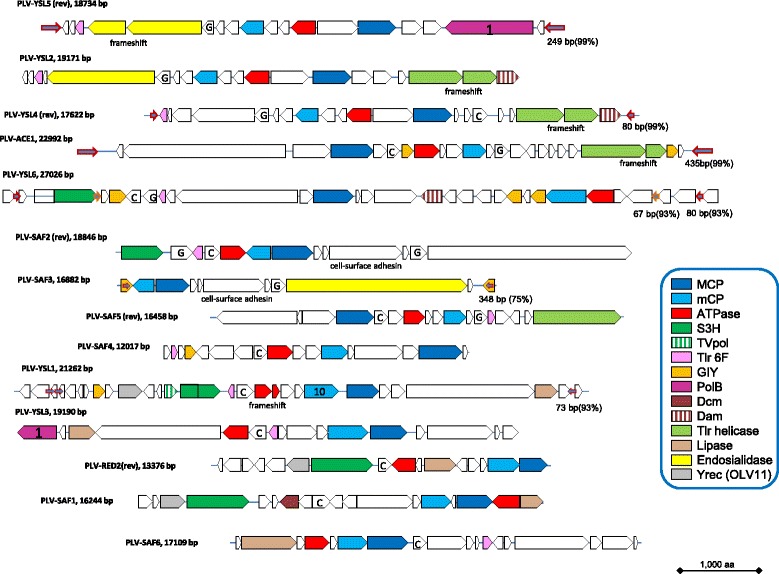
Genome architectures of the Polinton-like viruses: genomic contigs extracted and assembled from metagenomes, the PgVV-like group. Tlr1 helicase, a superfamily 1 helicase similar to the helicase encoded by the Polinton-like element Tlr1 from *Tetrahymena*. The other designations and color coding are the same as in Fig. 1

**Fig. 3:**
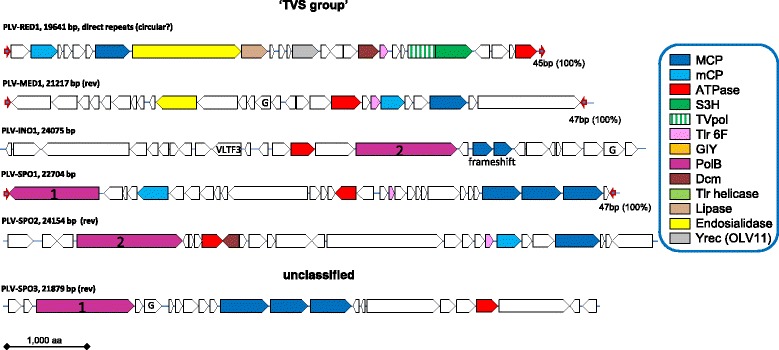
Genome architectures of the Polinton-like viruses: genomic contigs extracted and assembled from metagenomes, the TVS-like group and unclassified contigs. The designations and color coding are the same as in Fig. 1

Comprehensive analysis of the putative proteins encoded by the genes in the neighborhoods of the detected PgVV-like MCP genes involved PSI-BLAST search against the nr database, HHpred search against the Pfam and Interpro databases, as well as custom profiles for poorly conserved virus proteins, e.g. mCP. These searches resulted in the determination of the evolutionary provenance and functional predictions for many genes and suggest the existence of a broad range of diverse Polinton-like viruses (PLV) (Figs. 1, 2 and 3). Many of the PLV contigs contain long TIR, resembling the genome structure of PgVV (Figs. 1, 2 and 3), suggesting that these are complete genomes within the polintovirus and virophage genome size range, namely between 18 and 28 kb. Notably, one PLV contig contains long direct terminal repeats suggestive of a circular genome (Fig. 3). Four of the PLV genomes are integrated in sequenced algal genomes, as indicated by the identification of sequences spanning the junctions between the putative viral and host genomes. Three such elements were detected in the *G. theta* genome and one in the genome of *M. neglectum* (Fig. 1). Notably, another PLV genome belongs to a poorly characterized spherical virus that has been isolated from the marine algae *Tetraselmis viridis* and *T. striata* [35–37]. The rest of the PLV genomes are assembled metagenomic contigs and thus their hosts cannot be taxonomically assigned.

The PLV were detected in samples from various locations and filtering fractions but primarily in the virus fraction, i.e. below 0.22 micrometer (see Additional file 2). Importantly, although the assembly was performed “blindly”, i.e. from any segments present in the metagenomic database, nearly all of the assembled genomes of the putative PLV consisted of sequences originating from the same sampling location (Additional file 2), compatible with the validity of the assembly. Therefore, we named the PLV according to the sampling location: ACE, Ace Lake (Antarctica); INO, Indian Ocean; MED, Mediterranean; RED, Red Sea; SAF, South Africa; SPO, South Pacific Ocean; and YSL, Yellowstone Lakes.

By the design of the search procedure, all PLV genomes encode an MCP. Several of the PLV show duplication or even triplication of the MCP gene that previously has not been detected in Polintons or virophages (Figs. 2 and 3) but has been observed in mimiviruses [38]. In addition, most of the PLV genomes, and in particular, all that contain TIR, suggestive of completeness, encode a mCP and a packaging ATPase (Figs. 1, 2 and 3). These three genes represent the (nearly) universal core of PLV genes that encode components of the virus morphogenetic module. The identification of the mCP merits special comment. The mCP sequences are extremely poorly conserved so that the prediction of the mCP in Polintons required multiple, exhaustive database searches. Even then, no mCP has been identified in PgVV. However, multiple PSI-BLAST iterations with all the predicted proteins of PLV yielded several connections with the predicted mCP of Polintons. For example, a PSI-BLAST search initiated with the sequence of a hypothetical protein encoded by gene 7 of *G. theta* element 2 showed a statistically significant similarity (e-value <0.001) to the predicted mCPs of several Polintons, as well as “Megavirales” starting with the second search iteration. The proteins with significant similarity to the predicted Polinton mCP are conserved in nearly all PLV (Figs. 1, 2 and 3). Taken together, these observations indicate that (nearly) all PLV encode both MCP and mCP.

Notably, none of the PLV encodes the capsid maturation protease that is present in all virophages and nearly all Polintons. Apart from the three core genes, the gene distribution in the PLV is patchy (Figs. 1, 2 and 3). Several PLV encode a pDNAP but only the integrated element from *M. neglectum* encodes a RVE integrase. Thus, in general, the PLV do not qualify as Polintons in which the DNAP and RVE integrase are universal signatures. The element from *M. neglectum* seems to be the only exception as it encodes both pDNAP and RVE, and thus could be considered a Polinton although its MCP is clearly more similar to those of the PLV.

Several other genes of the PLV are shared with subsets of Polintons and/or virophages including putative primase-superfamily 3 helicase, superfamily 1 helicase, GIY-YIG endonuclease, lipase and two uncharacterized genes, Tlr6f and Organic Lake virophage gene 11 product, OLV11 (Figs. 1, 2 and 3). Unexpectedly, our database searches showed that the OLV11 protein that is conserved in several of the PLV and the Organic Lake group of virophages is a member of the tyrosine recombinase superfamily that is extremely widespread in bacteria, archaea and their viruses, as well as bacterial and some eukaryotic transposons [39] (Additional file 3). OLV11-like recombinases are also encoded by PgVV and Aureococcus anophagefferens virus (gi|672551235; 2nd PSI-BLAST iteration, E = 2e-04), a member of the “Megavirales”. The three elements integrated in the *G. theta* genome encode a distinct subgroup of tyrosine recombinases (Fig. 1). Finally, Sputnik-like virophages encode tyrosine recombinases [25] that are not recognizably similar to either OLV11-like predicted recombinases or the recombinases encoded by the *G. theta* PLV. These findings suggest that different PLV, virophages and Polintons employ unrelated or distantly related enzymes for integration into the host genomes and have acquired the corresponding recombinase genes on multiple, independent occasions. A new common theme among the PLV is the presence of genes encoding predicted DNA methylases of the Dam and Dcm families in several genomes (Figs. 1, 2 and 3).

Of special note is the abundance of genes encoding diverse lipases in PLV and virophages. In particular, we identified a previously unnoticed putative lipase in the genome of PgVV (ORF6; HHpred hit to cd00519, Lipase_3, Probability = 96.8). Notably, some Polintons encode lipases most closely related to the PLA2 phospholipase domain that is tethered to the capsid proteins of parvoviruses. In a close parallel to the recombinases, these lipase genes appear to have been acquired independently on several occasions. By analogy with parvoviruses that employ the lipase activity to disrupt the cellular endosomal membrane during viral entry [40, 41], it seems likely that the lipases of the PLV, Polintons (polintoviruses) and virophages are packed into the virions and facilitate virus penetration into the host cells.

The small protein Tlr6f is conserved in nearly all PLV, several virophages, numerous members of the “Megavirales” and some poorly characterized phages, suggestive of an important role in the reproduction of diverse viruses, but shows no detectable similarity to any domains with known structure or function. Another uncharacterized gene, provisionally denoted G in Figs. 1, 2 and 3, encodes a small protein without detectable similarity to any characterized domains that is conserved in a variety of PLV and also many phycodnaviruses and bacteria (Additional file 3).

### Evolutionary relationships of the PLV genes

**Fig. 4:**
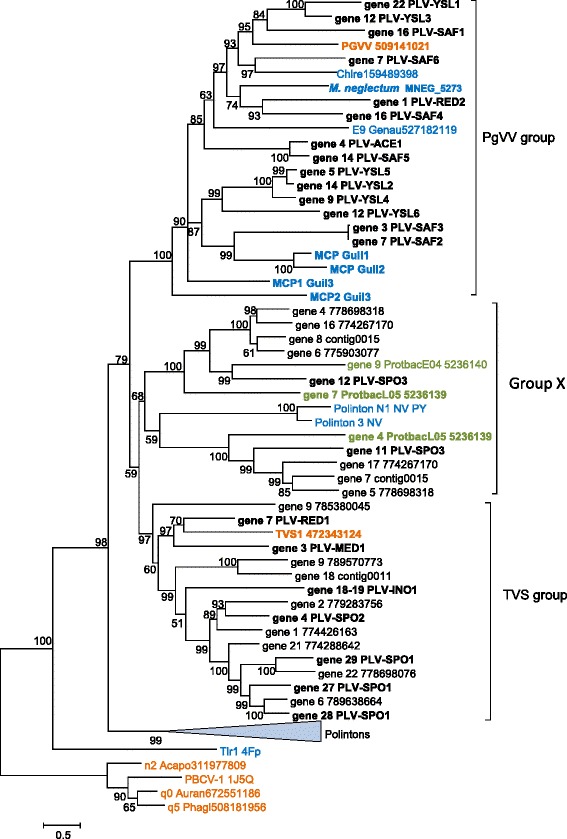
Phylogenetic tree of the major capsid proteins of Polinton-like viruses and related integrated elements. Proteins shown in Figs. 1, 2 and 3 are marked in bold. Color code: blue, eukaryotes; green, bacteria; orange, viruses; and black, metagenomes. For protein sequences present in GenBank, the species name abbreviation and the protein identification numbers are indicated; sequences translated in this work are marked as “gene xx” followed by either contig number (for in-house assemblies) or nucleotide identification number (for GenBank metagenomic assemblies). Acapo, *Acanthamoeba polyphaga* mimivirus; Auran, *Aureococcus anophagefferens* virus; Chlre, *Chlamydomonas reinhardtii*; Genau, *Genlisea aurea*; Guil1, Guil2, Guil3, *Guillardia theta* elements 1, 2, 3, respectively; NV, *Nematostella vectensis*; PBCV-1, *Paramecium bursaria* Chlorella virus type 1; PGVV, *Phaeocystis globosa* virus virophage; Protbac, *Proteobacteria bacterium* JGI 0000113; TVS1, *Tetraselmis viridis* virus S1

In an attempt to get insights into the evolution of the PLV, we analyzed phylogenetic trees for the MCP, the packaging ATPase and the pDNAP (the sequence conservation for the mCP is too low to produce a reliable phylogeny). The MCP sequences of the PLV were supplemented with a representative sample of the “solitary” MCPs that were detected in our search of the metagenomic and genomic sequences, and subjected to phylogenetic analysis jointly with a selection of the Polinton MCPs that appear to be the closest homologs identified in database searches (see Additional files 3 and 4). The tree was rooted using the MCP sequences of phycodnaviruses and a mimivirus, apparently the next closest family [21], as the outgroup. The resulting phylogenetic tree contains two major clades, one of which can be denoted the PgVV group and the other one the TVS group (Fig. 4), after the best characterized representative, *Tetraselmis viridis* virus S1 (TVS1; Fig. 3). The PgVV-like group includes all the PLV sequences shown to be integrated into algal genomes, as well as several solitary MCPs identified in genomic sequences from plants and algae. A third clade of the PLV (group X in Fig. 4) is weakly affiliated with the TVS group. The monophyly of the PLV with respect to Polintons is moderately supported except that two Polintons from the sea anemone fall within the X group of PLV (Fig. 4). These are the same Polinton MCPs that showed the highest similarity to the putative MCP of PgVV in the previous analysis [21]. The respective Polintons might be chimeric elements encoding a PLV-type MCP along with pDNAP and RVE integrase characteristic of Polintons.

**Fig. 5:**
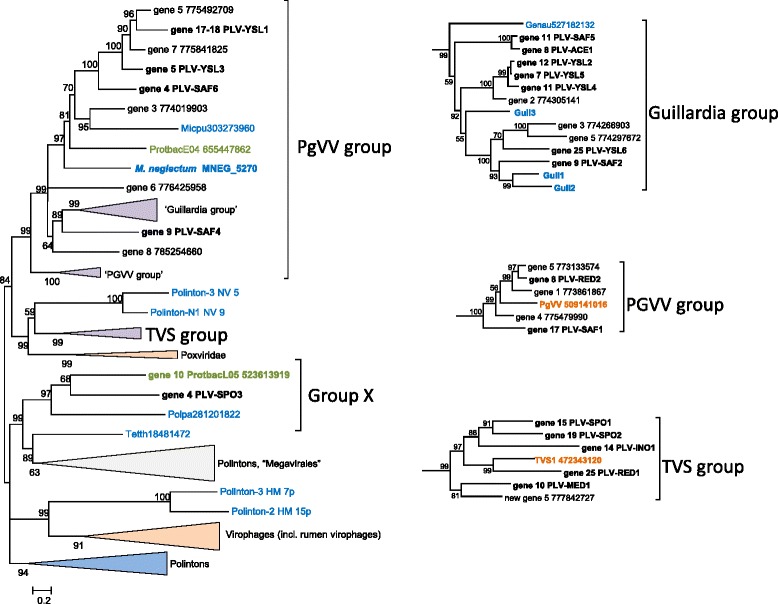
Phylogenetic tree of the maturation ATPases of Polinton-like viruses, Polintons, virophages and related elements. Branches with bootstrap support less than 50 were collapsed. Sequence labeling is the same as on Fig. 4. Genau, *Genlisea aurea*; Guil1, Guil2, Guil3, *Guillardia theta* elements 1, 2, 3, respectively; HM, *Hydra magnipapillata*; Micpu, *Micromonas pusilla*; NV, *Nematostella vectensis*; PGVV, *Phaeocystis globosa* virus virophage; Polpa, *Polysphondylium pallidum*; Proba, *Proteobacteria bacterium* JGI 0000113-E04; Tetth, *Tetrahymena thermophila*; TVS1, *Tetraselmis viridis* virus S1; TVS, *Tetraselmis viridis* virus S1 group

The phylogenetic tree of the packaging ATPases provides for inclusion of homologs from diverse sources, in particular Polintons, virophages and members of the “Megavirales” [22]. However, the resolution power of the phylogenetic analysis in this case is low because the multiple alignment of the ATPases underlying the tree includes only 165 phylogenetically informative positions (see Additional files 3 and 4). The topology of the ATPase tree is generally compatible with the MCP phylogeny in that both the PgVV and TVS groups come across as monophyletic (Fig. 5). However, the monophyly of all PLV was not recovered because the members of the Group X of PLV clustered with Polintons and members of the “Megavirales”, and in addition, the TVS group clustered with poxviruses (Fig. 5). It remains unclear whether these affiliations in the ATPase tree reflect independent acquisition or replacement of this gene in different groups of PLV or are due to long-branch attraction and other phylogenetic artifacts. Despite the consistent segregation of PgVV-like and TVS-like groups of PLV in phylogenetic analyses, the gene repertoires of the two groups are closely similar indicative of the coherence of the PLV as a whole (Figs. 2 and 3).

**Fig. 6:**
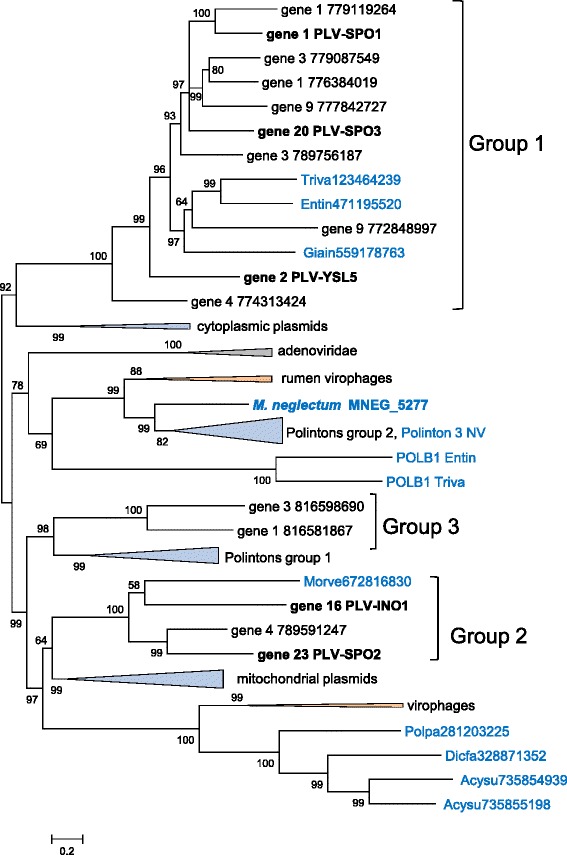
Phylogenetic tree of protein-primed DNA polymerase Polinton-like viruses, Polintons, virophages and related elements. Sequence labeling is the same as in Fig. 4. Acysu, *Acytostelium subglobosum*; Dicfa, *Dictyostelium fasciculatum*; Entin, *Entamoeba invadens*; Giain, *Giardia intestinalis*; HM, *Hydra magnipapillata*; Micpu, *Micromonas pusilla*; Morve, *Mortierella verticillata*; NV, *Nematostella vectensis*; Polpa, *Polysphondylium pallidum*; Triva, *Trichomonas vaginalis*

As observed previously for virophages and Polintons [29], analysis of the PLV genes implicated in genome replication revealed complicated relationships suggestive of complex evolution. The pDNAPs of the PLV form three distinct clades (Fig. 6; see Additional files 3 and 4). The largest of these, denoted Group 1 in Fig. 6, clusters with the pDNAPs of fungal cytoplasmic DNA plasmids. Group 2 is associated with the pDNAPs of mitochondrial plasmids, Mavirus-like virophages and a distinct subfamily of Polintons, whereas Group 3 is the sister group of the Polinton group 1 clade (Fig. 6). Finally, the pDNAP of the *M. neglectum* element belongs to the Polinton group 2 rather than any of the PLV clades (Fig. 6). Notably, the pDNAPs of Group 1 are represented both in the PgVV-like group and the TVS-like group of the PLV (Figs. 2 and 3). Each of these affinities has a strong bootstrap support (Fig. 6). Thus, it appears most likely that the PLV acquired the pDNAP genes from Polintons and possibly from DNA plasmids on multiple occasions, resulting in the combination of distinct DNAPs with different morphogenetic modules.

Apart from the pDNAPs, several PLV encompass the gene for a superfamily 3 helicase (S3H) that in PgVV, PLV-YSL1 and PLV-RED1 is fused to a distinct homolog of bacterial DNA polymerase I, known as TVpol (transposon-virus polymerase). A homologous fusion protein is encoded by Sputnik and other virophages and is predicted to function as the primase-helicase in genome replication [42]. Several other PLV encode S3H that is not directly related to that in the TVpol fusion proteins and is likely to have an independent origin (Figs. 2 and 3). In two of the three *G. theta* integrated elements, the N-terminal regions of the respective proteins are typical archaeo-eukaryotic primases, so that the entire protein has the same domain architecture as the primase-helicase of the “Megavirales”. In the rest of the PLV, the region upstream of S3H lacks detectable homologs and potentially could encompass divergent primases or inactivated derivatives thereof.

## Discussion

The PLV comprise of a diverse set of putative viruses that is defined by the distinct, PgVV-like MCP and universally share three genes, those for the MCP, mCP (with a few uncertainties) and the packaging ATPase. It should be noted that of the numerous PgVV-like MCP detected in the present analysis, less than 10 % were found in large or extendable contigs and thus were analyzed here in detail. Altogether, the PLV seem to be an abundant group of viruses that remains to be characterized through a combination of metagenomic and virological approaches.

**Fig. 7:**
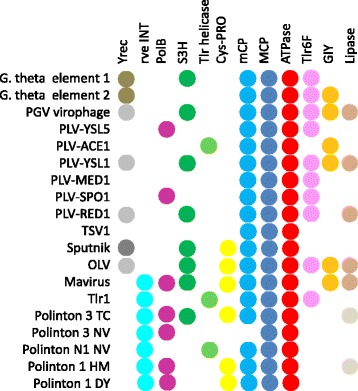
Shared genes between Polinton-like viruses, Polintons and virophages. Different shades in the same column denote distantly related proteins that most likely have been acquired independently

The conserved genes of the PLV represent the minimal morphogenetic module that is shared with many other viruses, including Polintons, virophages, adenoviruses and the “Megavirales”, and in most of these eukaryotic viruses, additionally includes the maturation protease. This protease is conspicuously missing from the PLV. Apart from the minimal morphogenetic module, subsets of the PLV share additional genes, some of which are implicated in genome replication, with each other, and also with Polintons and virophages (Fig. 7).

The discovery of the PLV expands the emerging class of dsDNA viruses of eukaryotes that share several distinctive characteristics: genome size of 15 to 30 kb; icosahedral particles approximately 60 nm in size; and homologous morphogenetic modules that consist of MCP, mCP, packaging ATPase and maturation protease [22, 24]. The morphogenetic modules can be reduced as is the case of the RVP, which lack the mCP [32], and the PLV which do not encode the protease. The typical Polintons (polintoviruses) also encode pDNAP and RVE integrase and so far have been found only in the integrated state. However, the finding that Polintons encode a MCP that, according to homology modeling results, retains the typical double jelly-roll structure, as well as the mCPs, strongly suggests the existence of Polinton (polintovirus) virions [21, 22]. As described here, several PLV are integrated into the host genomes but only one of the integrated elements encodes a RVE integrase. This genome organization of this element integrated into the genome of the alga *M. neglectum* actually resembles a *bona fide* Polinton more than a typical PLV. The remaining integrated PLV lack any enzymes that could be implicated in integration and could conceivably employ integrases of resident Polintons within the same host *in trans*. In contrast, several other PLV encode the newly identified OLV11-like tyrosine recombinase and thus can be predicted to lead a dual, Polinton-like life style combining a virus stage with an integrated stage, similar to the Polintons.

The involvement of the OLV11-like tyrosine recombinase in the integration of the PLV into the host genome is compatible with the evidence that PgVV integrates into the genome of its helper virus, PGV [34]. In this work, we identified a protein, the OLV11-like tyrosine recombinase encoded by PgVV open reading frame (ORF) 3, which is likely to be responsible for this integration. Notably, the recombination hotspot on the PgVV genome has been mapped to the region between ORF3 and ORF4 [34], pinpointing the location of a putative attachment site on the virophage genome. In this respect, PgVV resembles many temperate bacterial and archaeal viruses in which the attachment sites are located next to the integrase genes [43]. In integration reactions mediated by tyrosine recombinases, the donor DNA molecule is typically circular [44], suggesting that the PgVV genome, and by extension the genomes of other PLV that encode the putative tyrosine recombinase, circularize prior to integration.

The divergent recombinases encoded by the three elements integrated into the genome of *G. theta* are of further interest. In all these elements, the recombinase genes are also located close to the extremities of the integrated genome, i.e. in the proximity of the attachment sites. Notably, in one of the elements, the recombinase gene is disrupted by the insertion of a Copia-like LTR-retrotransposon (Fig. 1), potentially leading to immobilization of this element or making it dependent on the supply of the integrase *in trans*. Given that the majority of the PLV were discovered in the virus fraction of the respective metagenomes and often lack a detectable integrase, many if not most of the PLV genomes likely originate from virus particles. The ability of the PLV to form virions is demonstrated by the fact that the experimentally characterized TVS1 belongs to this group [35–37].

Typical of metagenome mining studies, the hosts of the PLV are unknown. Nevertheless, the integration of several PLV genomes into algal genomes, together with the fact that the only experimentally characterized virus among the PLV, TVS1, infects an alga [35], imply that most if not all PLV are algal viruses. The association of the PgVV with a virus that belongs to the extended family *Mimiviridae* [34] suggests the possibility that some or all of the PLV parasitize on large viruses, i.e. represent a distinct variety of virophages. Integration of the Sputnik and PgVV virophages into the host virus genomes has been reported [34, 45].

The morphogenetic module and the DNAPs of Polintons appear to originate from the homologous module of bacteriophages of the family *Tectiviridae* [22]. However, tectiviruses lack the protease and the integrase that most likely have been acquired at a later stage of evolution, possibly, from a single Ginger-like transposon [22]. Thus, the PLV might resemble the ancestral forms of polintoviruses (Polintons). However, the alternative possibility, that the PLV are derived polintoviruses, cannot be ruled out. Moreover, given that the PLV mix with polintoviruses in some phylogenetic trees, in particular, the MCP tree (Fig. 4), multiple, independent origins of PLV from different groups of Polintons appear possible.

As shown previously, the RVP are chimeric viruses that combine the virophage morphogenetic module with the Polinton-derived pDNAP [32]. Here we demonstrate that different groups of PLV defined by phylogenetic analysis of the MCP and the packaging ATPase share the pDNAPs and other replicative enzymes with different groups of Polintons and virophages. Thus, some of the PLV also appear to be chimeras, an evolutionary trend that is increasingly observed in different groups of viruses [24, 46]. Moreover, the network-type relationship between the gene complements of the PLV, Polintons and virophages (Fig. 7) indicates that, on the evolutionary scale, they all share a common gene pool. Apparently, this gene pool has spawned a great variety of diverse genetic elements. The PLV are unlikely to be the last group of viruses in this class to be discovered.

## Conclusions

Metagenomic database mining increasingly leads to the discovery of novel groups of organisms. Identification of new viruses is particularly straightforward given the comparatively small size of viral genomes, but presents additional challenges due to the typical fast evolution of viruses resulting in difficulties with respect to the detection of homologous relationships. Here we report a comprehensive metagenomic database search complemented by the use of sensitive methods to detect homologous proteins that resulted in the discovery of a novel group of Polinton-like viruses. These putative viruses resemble Polintons (polintoviruses) in the overall genome organization but possess a distinct form of the major capsid protein and a minimal morphogenetic module lacking the maturation protease that is typical of Polintons and virophages. With a single exception, the PLV also lack the RVE integrase that is encoded in the genomes of all Polintons and the Mavirus group of virophages. However, several PLV encode a predicted novel tyrosine recombinase that could provide an alternative route of integration. Although we identified several PLV genomes and individual genes integrated into eukaryotic genomes, it appears likely that most of the PLV are viruses. Given the lack of protease and RVE integrase, which appear to be relatively late acquisitions during the evolution of Polintons, the PLV could resemble the ancestral polintoviruses that evolved from bacterial tectiviruses, although the possibility that the PLV are degraded derivatives of polintoviruses could not be ruled out either.

Apart from the conserved minimal morphogenetic module, the PLV widely differ in their gene repertoires but share a network of homologous genes with Polintons and virophages. Although we explicitly analyzed only 20 PLV genomes that could be assembled from metagenomic contigs to (near) completion, the overall number of detected PLV-type MCP is much greater indicating that these viruses are common, at least in marine habitats. To summarize, Polintons (polintoviruses), PLV and virophages are widespread among eukaryotes, share a common gene pool and appear to represent an emerging major class of eukaryotic viruses and transposons. Most likely, new families of viruses and other mobile elements within this class remain to be discovered.

## Methods

### Metagenomic database screening

The sequence of the PgVV MCP was first used as a query in a PSI-BLAST search [47] of the non-redundant protein sequence database (nr) at the NCBI. This search detected homologs of the PgVV MCP in five eukaryotic genomes. PgVV MCP and its eukaryotic homologs found in the nr database were used as queries for TBLASTN searches (e-value ≤10) against two metagenomic databases, CAMERA [48] and the NCBI Whole Genome Shotgun (WGS) contigs database [49].

After the metagenomic PLV fragments were assembled, translated and validated (as described below), a preliminary phylogenetic tree for all detected MCP-like proteins of PLV was constructed using FastTree [50]. Using this tree as a guide, 20 diverse representatives were selected, and the metagenomic databases were screened once again, as described above.

### CAMERA sequence assembly

Because the CAMERA database is mostly composed of unassembled reads, the hits obtained from CAMERA were assembled using Geneious Pro 8.0.2 (www.geneious.com), separately for each query. Resulting contigs were translated using GeneMark [51] and checked for presence of a PLV MCP-like protein, using either (i) first BLASTP nr hits of GeneMark-translated proteins, or (ii) PSI-BLAST initiated by a profile made from all MCPs detected up to that point against the GeneMark-translated proteins. The contigs encoding proteins with best hits to one of the identified MCPs or matching the MCP profile, were collected. Only contigs encoding PLV-like MCPs (hereinafter seeds) were taken to the next step.

For each seed, BLASTN search of the terminal regions (400 nt) against CAMERA was performed using MegaBLAST [52]. Highly similar (97 % identical nucleotides) hits were collected and assembled with the seed (Geneious Pro 8.0.3, *de novo* assembly algorithm). The resulting contig was used as a seed again, and the cycle was repeated until the contig could not be extended any longer. To validate and refine the final assembly, the last seed was searched against CAMERA using MegaBLAST, highly similar (97 % identical nucleotides) hits were collected and assembled using the last seed as a guide by Geneious (combined mapping and *de novo* assembly workflow). All final contigs were manually checked for assembly errors.

### WGS sequence assembly

The sequences obtained by the TBLASTN searches against the WGS database were collected and translated by GeneMark. Sequences not containing PLV-like MCPs were filtered out as described for CAMERA (above). All remaining sequences belonged to the marine metagenome subset of WGS metagenomic data, and accordingly, all subsequent BLASTN searches were performed against marine metagenomes. The collected metagenomic sequences were extended whenever possible using alternating cycles of BLASTN searches and assembly with Geneious. This procedure allowed for extension of some of the Tara Oceans sequences [7] that have been assembled prior to database submission.

### Protein sequence analysis

The sequences of contigs obtained as described above were translated using GeneMark, and the resulting protein sequences were used as queries to search the nr database using PSI-BLAST, the Conserved Domain Database (CDD) using RPS-BLAST [53], and the CDD and Pfam databases using HHpred [54].

### Phylogenetic analysis

The MCP, ATPase and pDNAP protein sequences collected previously [29] were pooled with the respective PLV proteins and their best hits from the nr database. The MCP sequences were aligned with PROMALS3D, with the PBCV MCP structure used as a template [55]. The ATPase and pDNAP sequences were aligned using MUSCLE [56]. Poorly aligned (low information content) positions were removed using the gappyout function of trimAl [57]. Phylogenetic trees were constructed using the PhyML program [58, 59], the latest version of which (http://www.atgc-montpellier.fr/phyml-sms/) includes automatic selection of the best-fit substitution model for a given alignment. The best models identified by PhyML were LG + G6 + I + F for MCP and ATPase, and Blosum62 + G6 + I + F for pDNAP. LG, Le-Gascuel matrix; G6 + I + F, gamma shape parameter: estimated; number of categories: 6; proportion of invariable sites: estimated; and equilibrium frequencies: empirical.

## Additional files

Additional file 1:
**Annotated genomes of Polinton-like viruses.** (TXT 748 kb) Additional file 2:
**Geographical origins of the PLV.** (XLSX 273 kb) Additional file 3:
**Multiple alignments of the core genes of the PLV.** (PPTX 1221 kb) Additional file 4:
**PhyML trees (Newick format), trimmed alignments used for phylogenetic tree construction, and original alignments for MCP, ATPase and pDNAP.** (TXT 510 kb)
