# Supplementary material for: A novel group of diverse Polinton-like viruses discovered by metagenome analysis
Source: BMC Biol. 2015 Nov 11;13:95. doi: 10.1186/s12915-015-0207-4 (PMC4642659; doi:10.1186/s12915-015-0207-4)
Supplement: Additional file 3: — Multiple alignments of the core genes of the PLV. (PPTX 1221 kb) [file 12915_2015_207_MOESM3_ESM.pptx]

## Slide 1
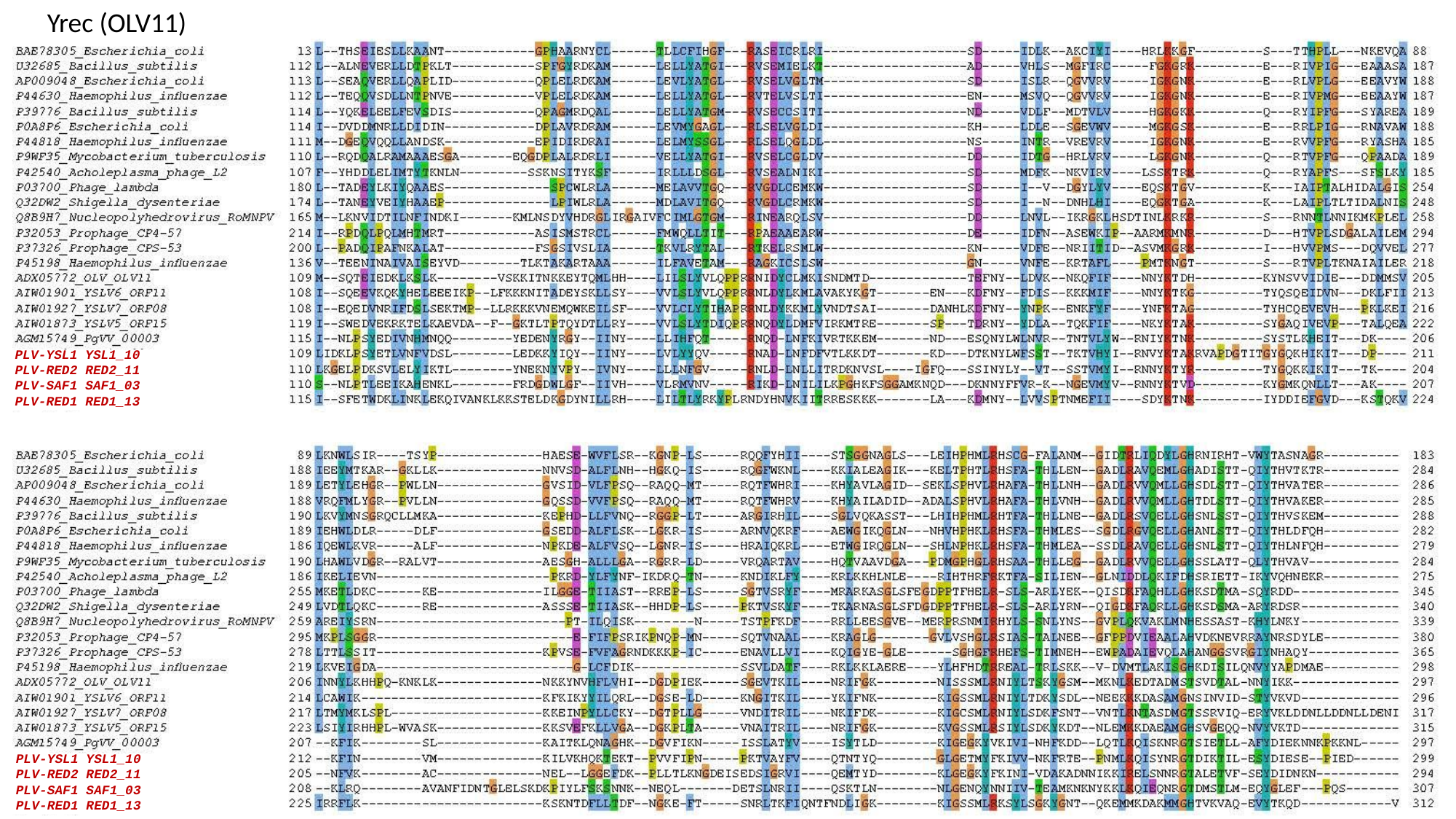

Yrec (OLV11)
PLV-YSL1 YSL1_10
PLV-RED2 RED2_11
PLV-SAF1 SAF1_03
PLV-RED1 RED1_13
PLV-YSL1 YSL1_10
PLV-RED2 RED2_11
PLV-SAF1 SAF1_03
PLV-RED1 RED1_13

## Slide 2
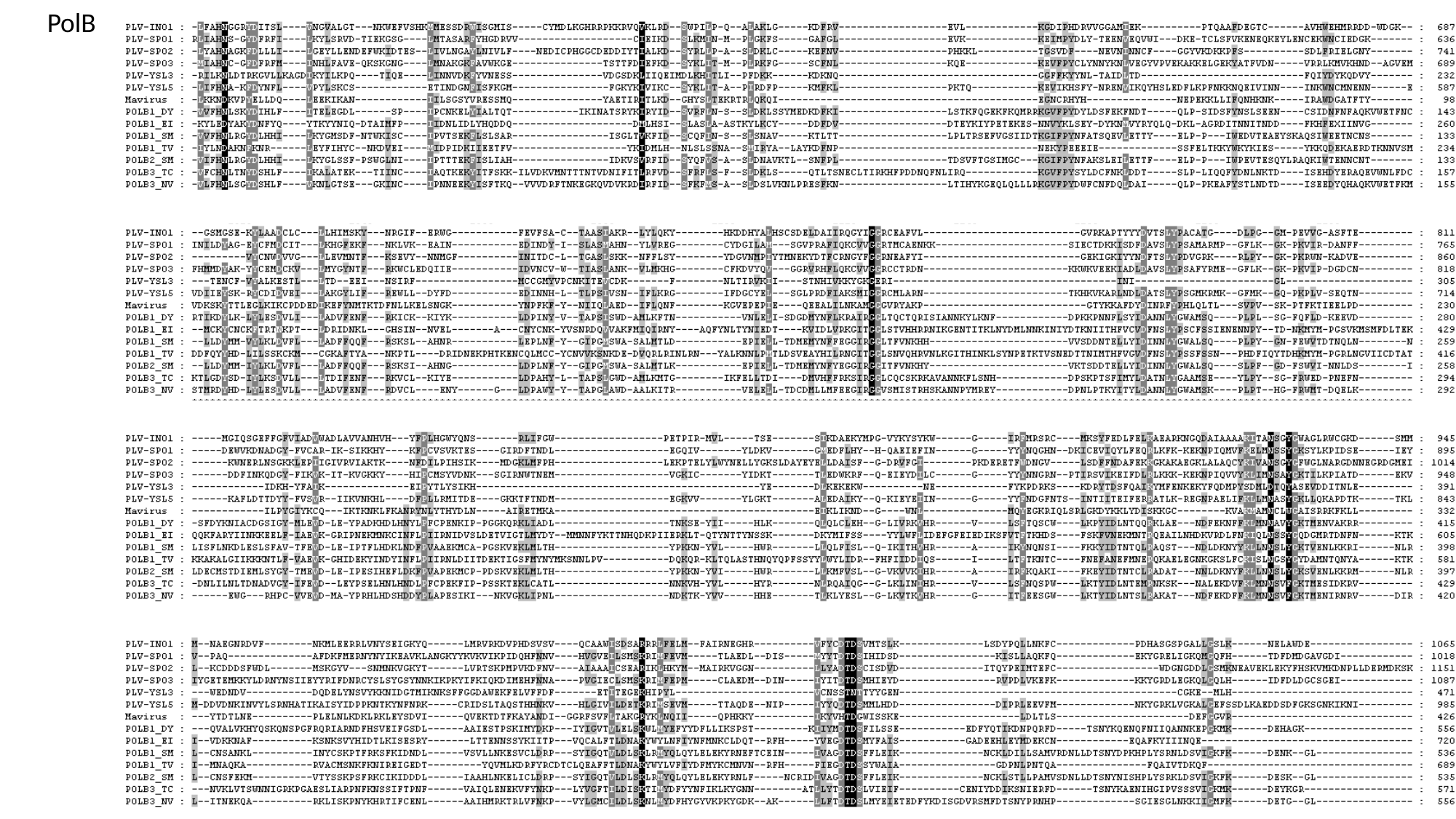

PolB

## Slide 3
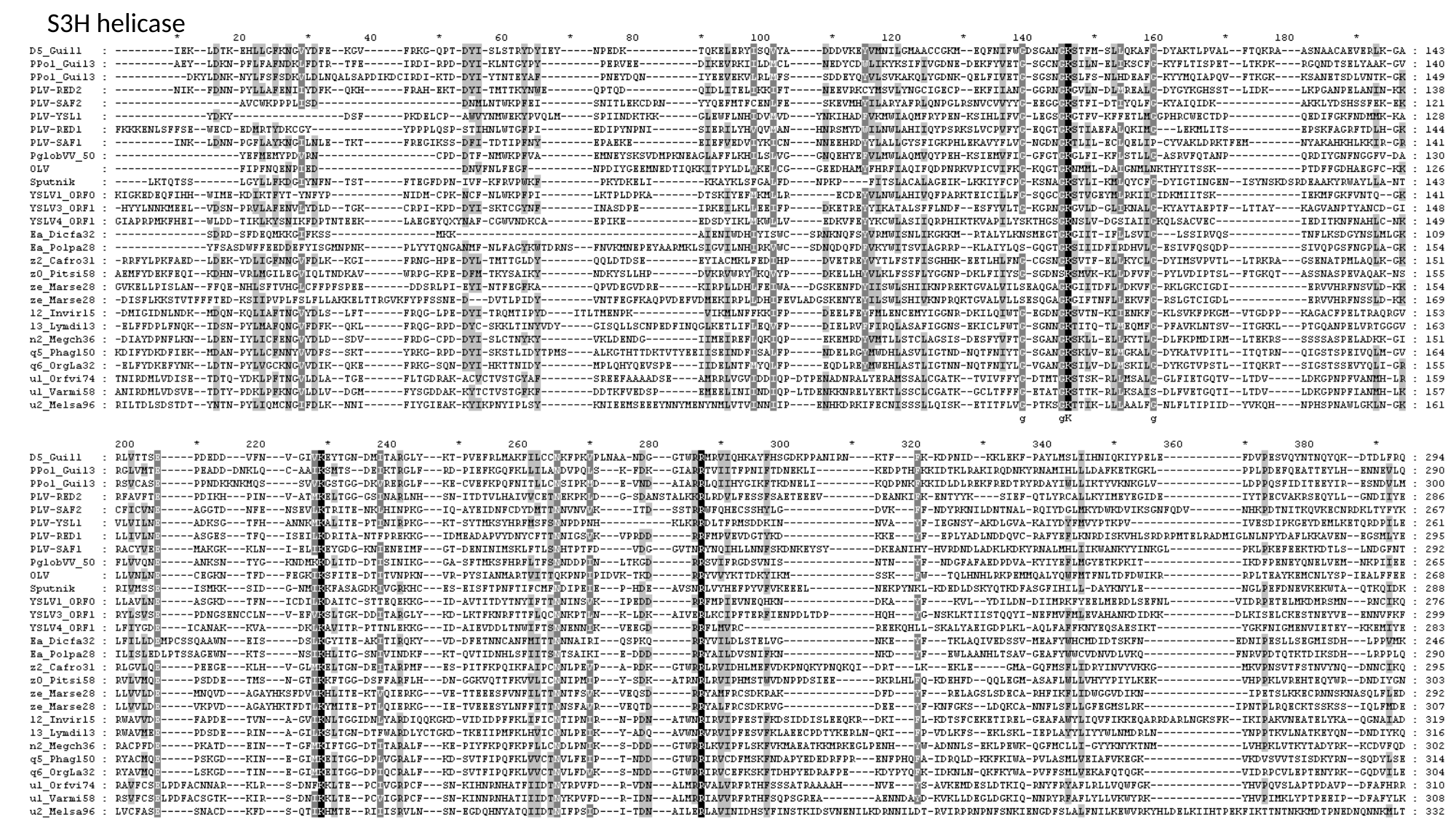

S3H helicase

## Slide 4
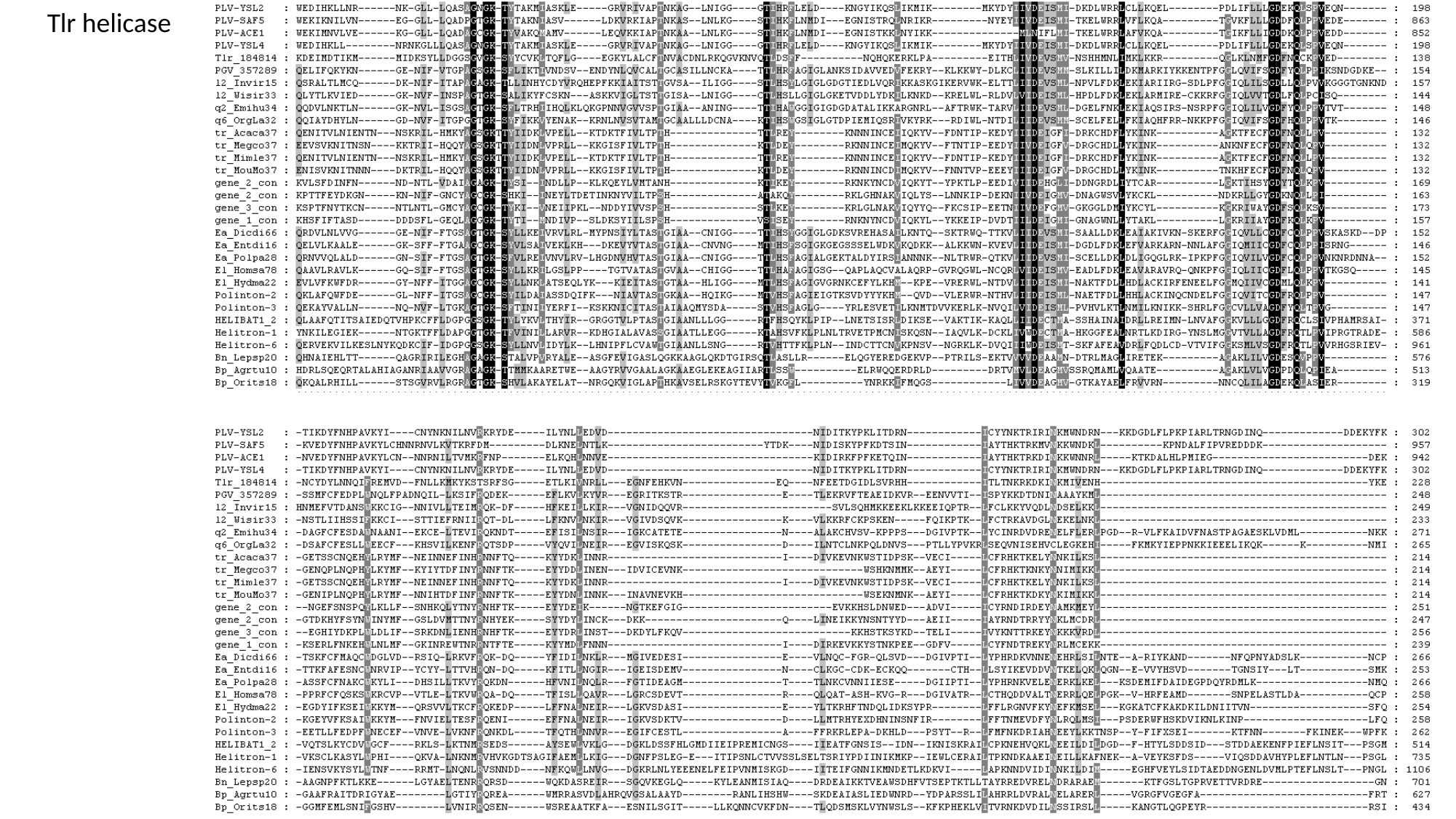

Tlr helicase

## Slide 5
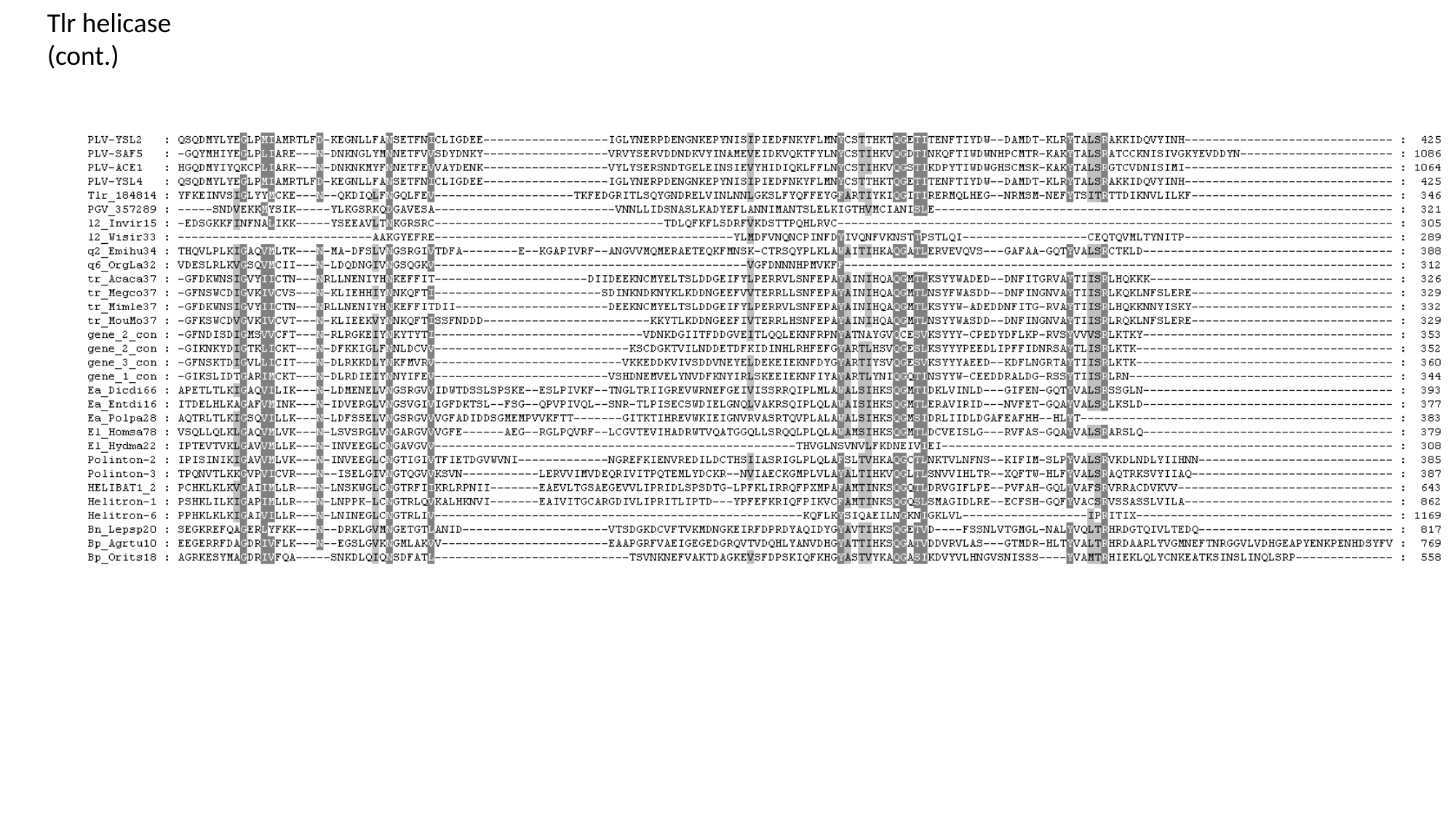

Tlr helicase (cont.)

## Slide 6
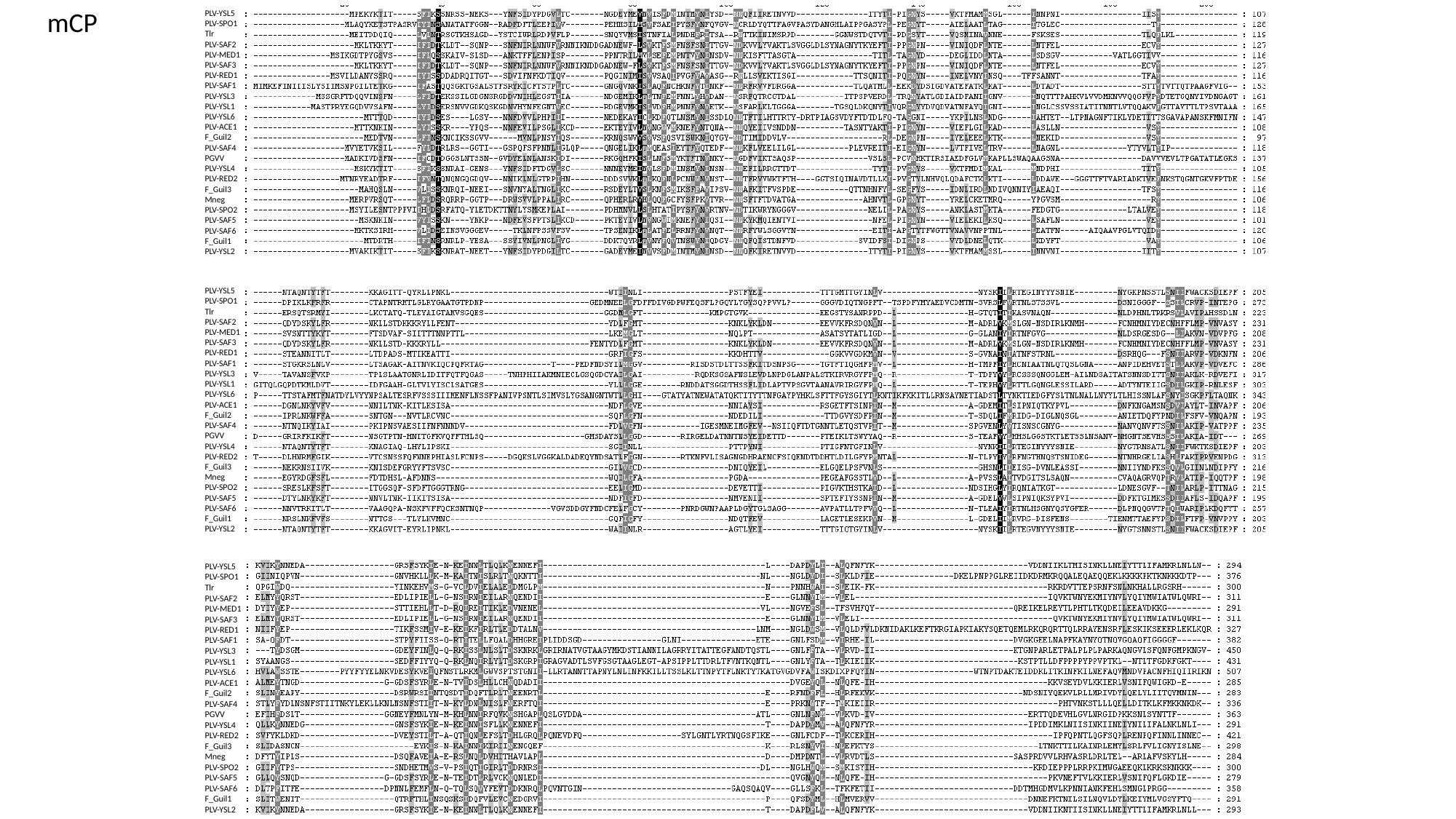

mCP
PLV-YSL5
PLV-SPO1
Tlr
PLV-SAF2
PLV-MED1
PLV-SAF3
PLV-RED1
PLV-SAF1
PLV-YSL3
PLV-YSL1
PLV-YSL6
PLV-ACE1
F_Guil2
PLV-SAF4
PGVV
PLV-YSL4
PLV-RED2
F_Guil3
Mneg
PLV-SPO2
PLV-SAF5
PLV-SAF6
F_Guil1
PLV-YSL2
PLV-YSL5
PLV-SPO1
Tlr
PLV-SAF2
PLV-MED1
PLV-SAF3
PLV-RED1
PLV-SAF1
PLV-YSL3
PLV-YSL1
PLV-YSL6
PLV-ACE1
F_Guil2
PLV-SAF4
PGVV
PLV-YSL4
PLV-RED2
F_Guil3
Mneg
PLV-SPO2
PLV-SAF5
PLV-SAF6
F_Guil1
PLV-YSL2
PLV-YSL5
PLV-SPO1
Tlr
PLV-SAF2
PLV-MED1
PLV-SAF3
PLV-RED1
PLV-SAF1
PLV-YSL3
PLV-YSL1
PLV-YSL6
PLV-ACE1
F_Guil2
PLV-SAF4
PGVV
PLV-YSL4
PLV-RED2
F_Guil3
Mneg
PLV-SPO2
PLV-SAF5
PLV-SAF6
F_Guil1
PLV-YSL2

## Slide 7
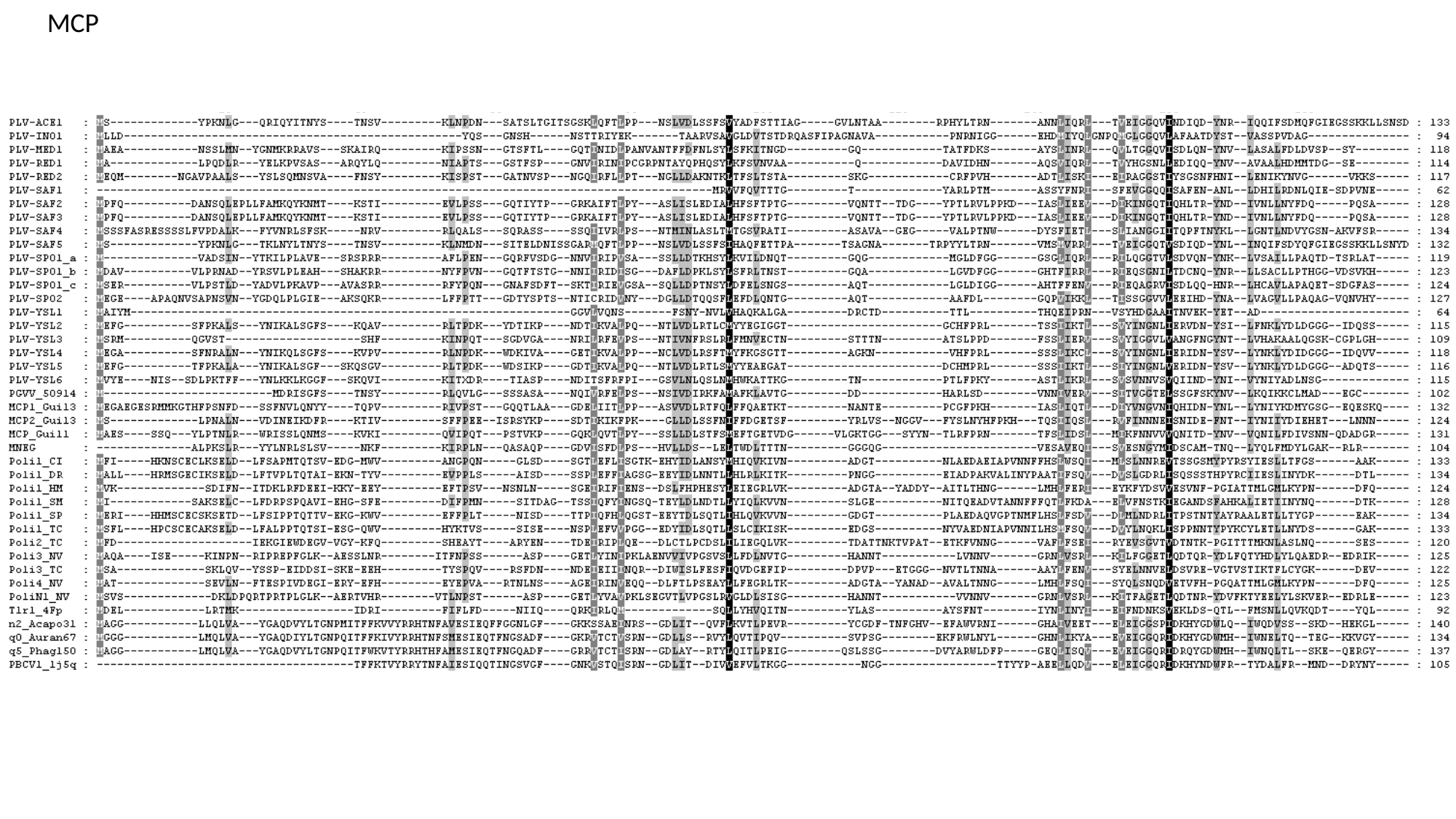

MCP

## Slide 8
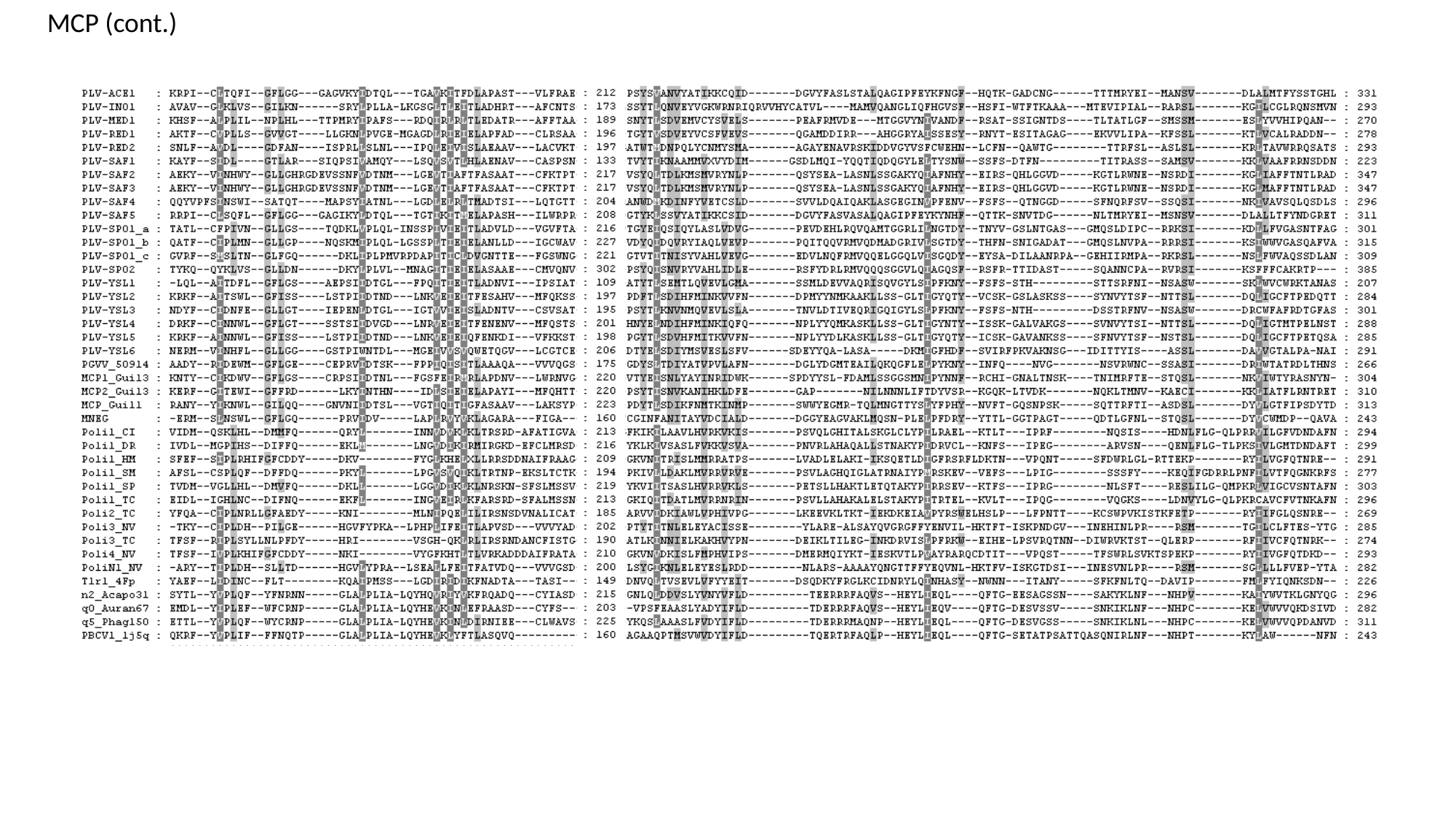

MCP (cont.)

## Slide 9
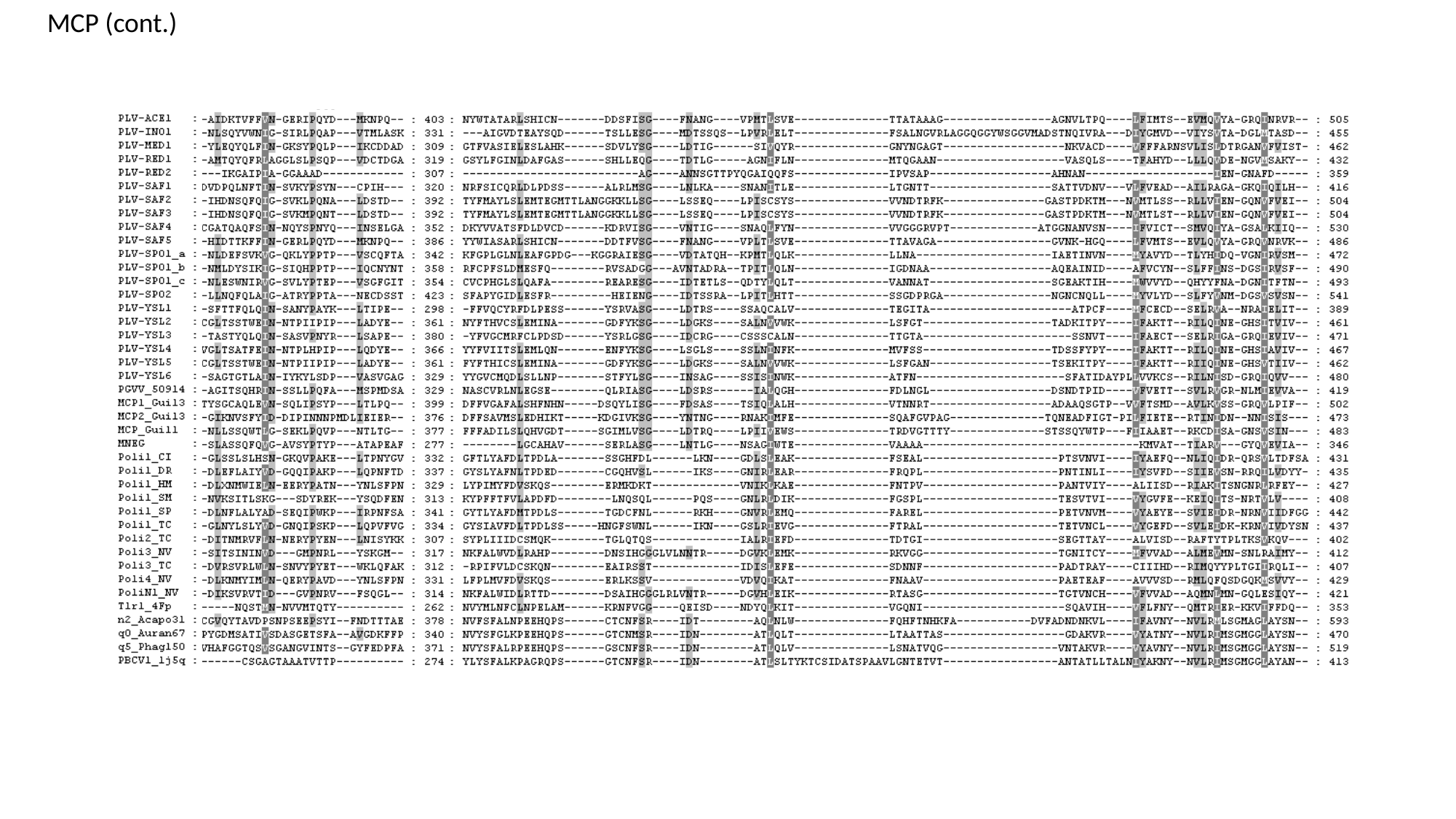

MCP (cont.)

## Slide 10
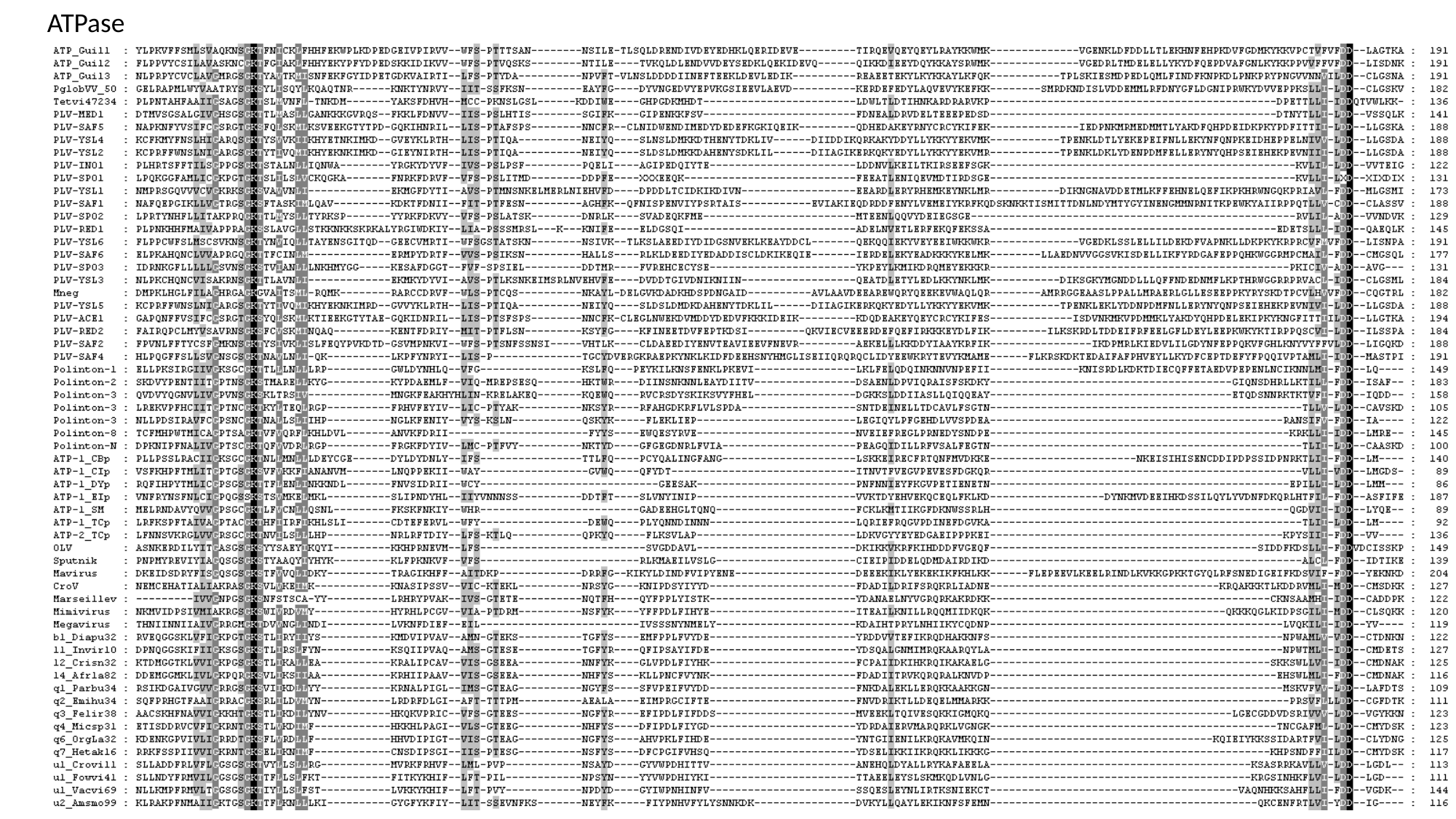

ATPase

## Slide 11
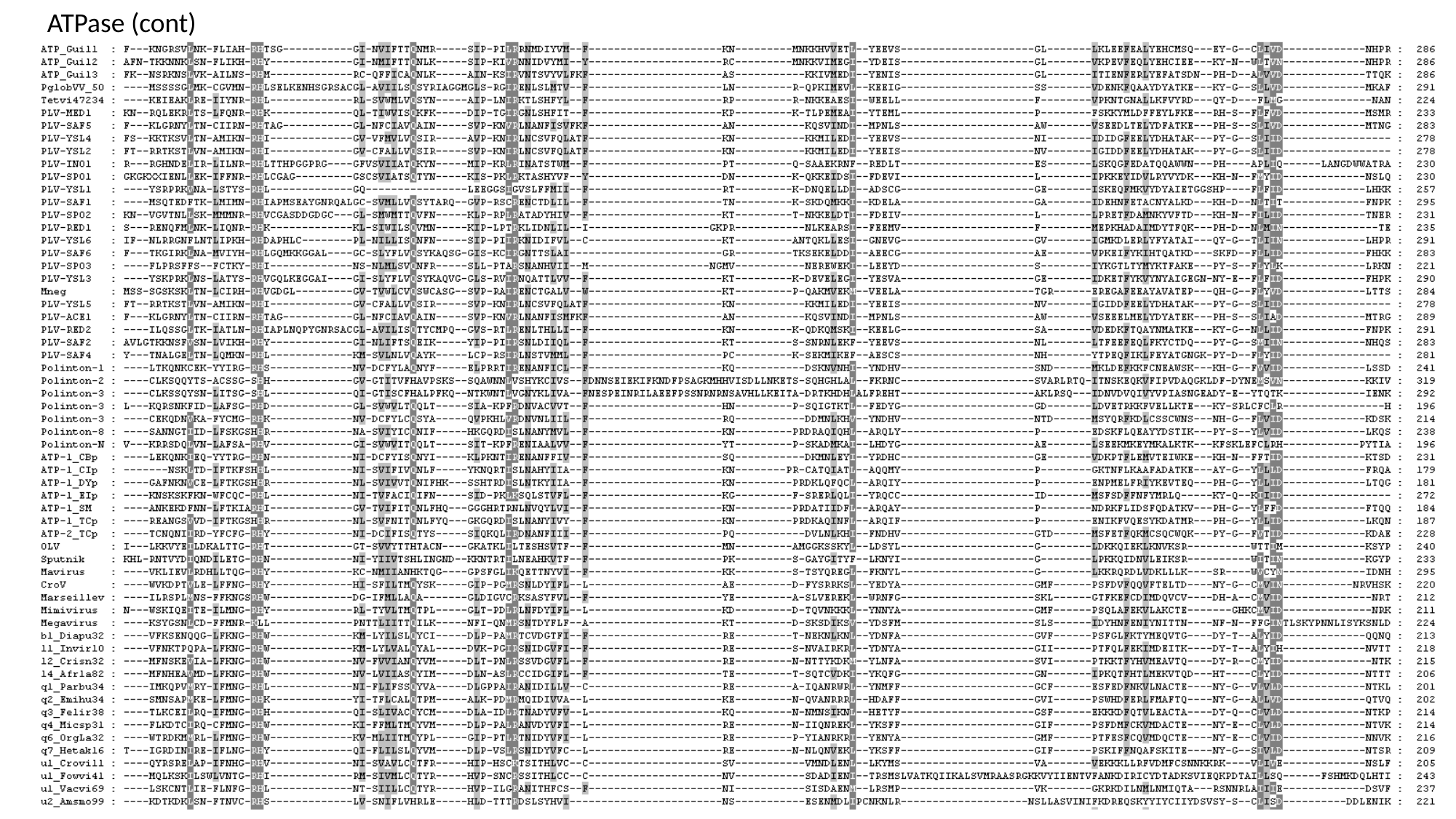

ATPase (cont)

## Slide 12
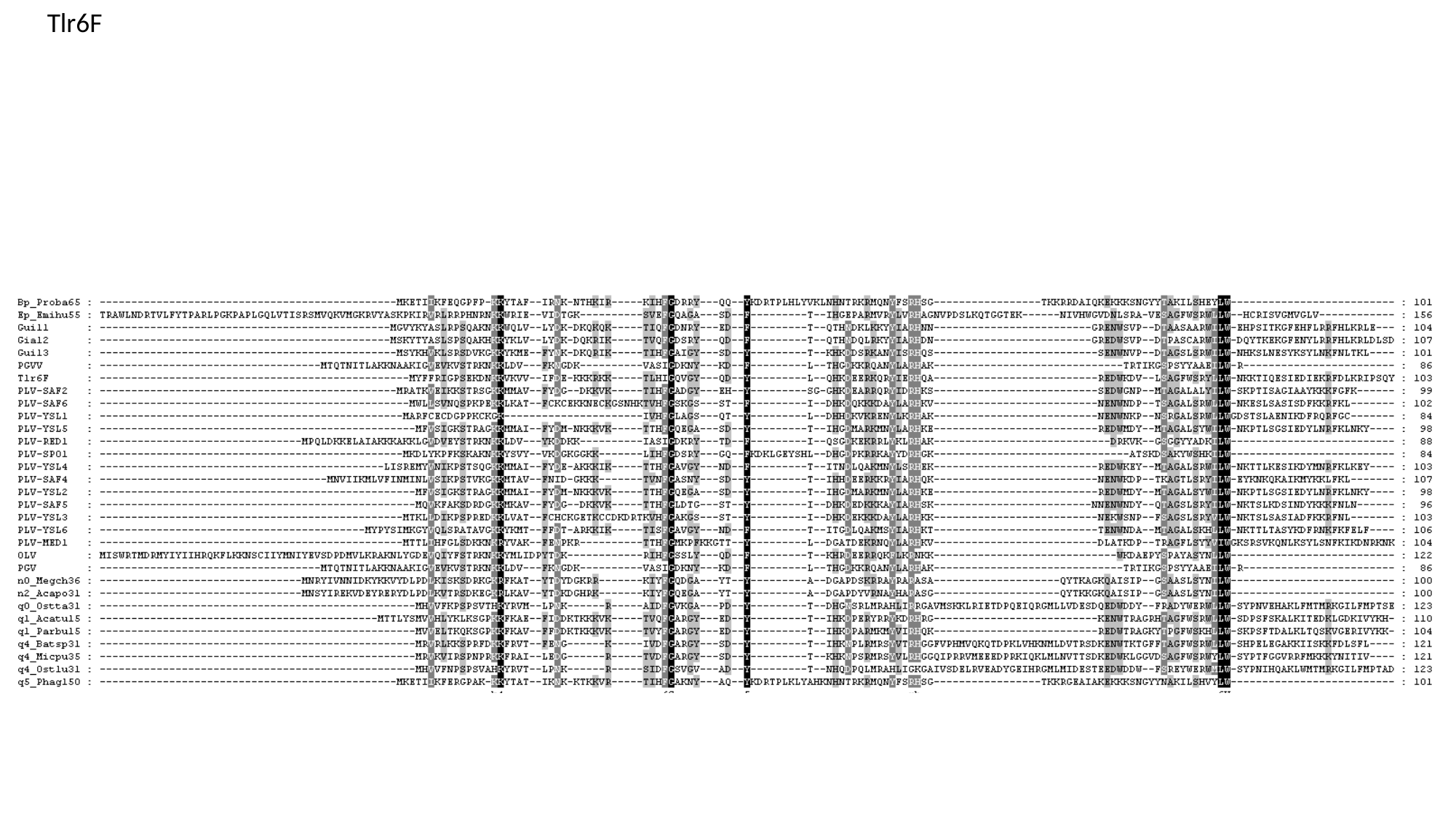

Tlr6F

## Slide 13
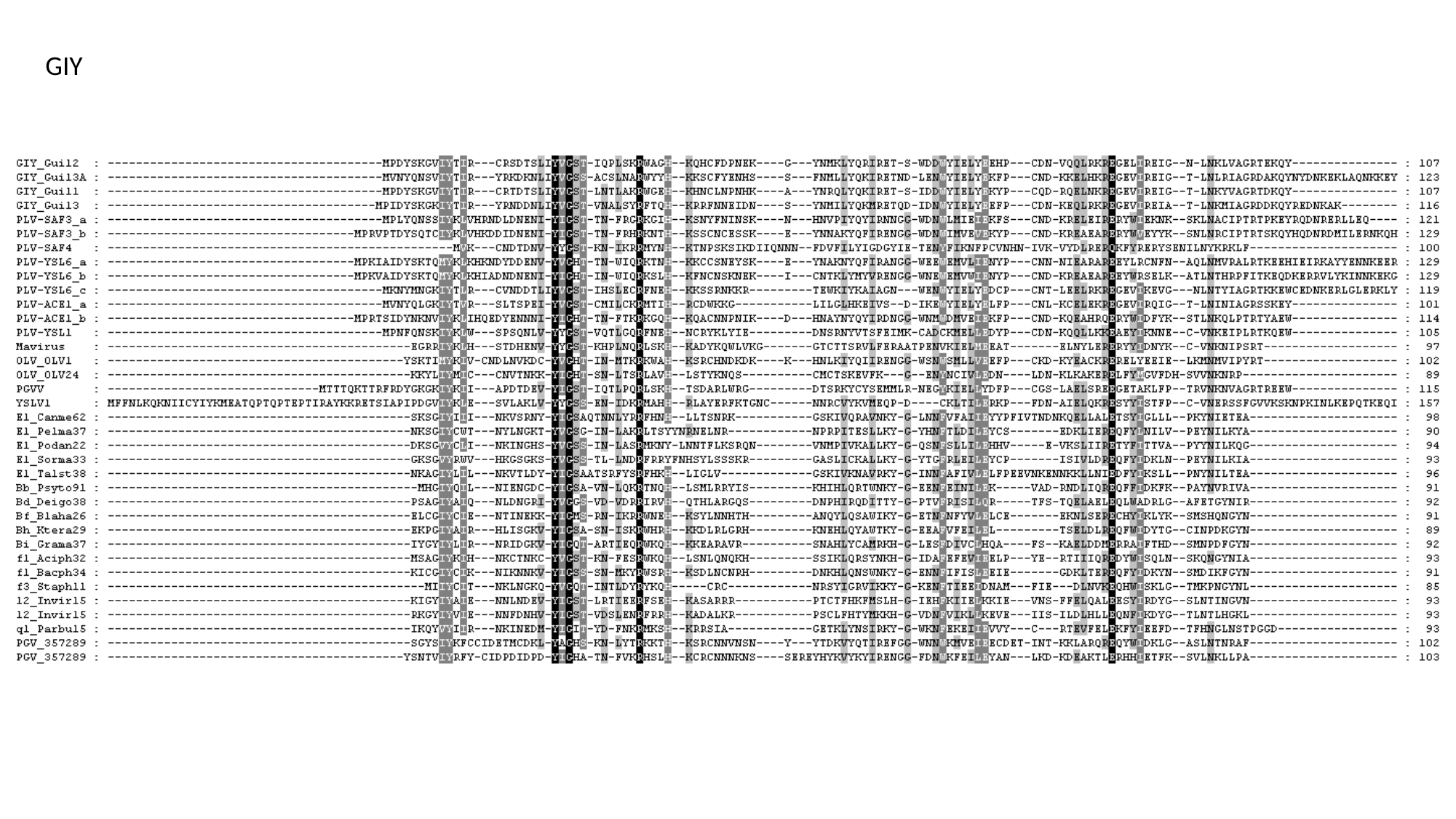

GIY

## Slide 14
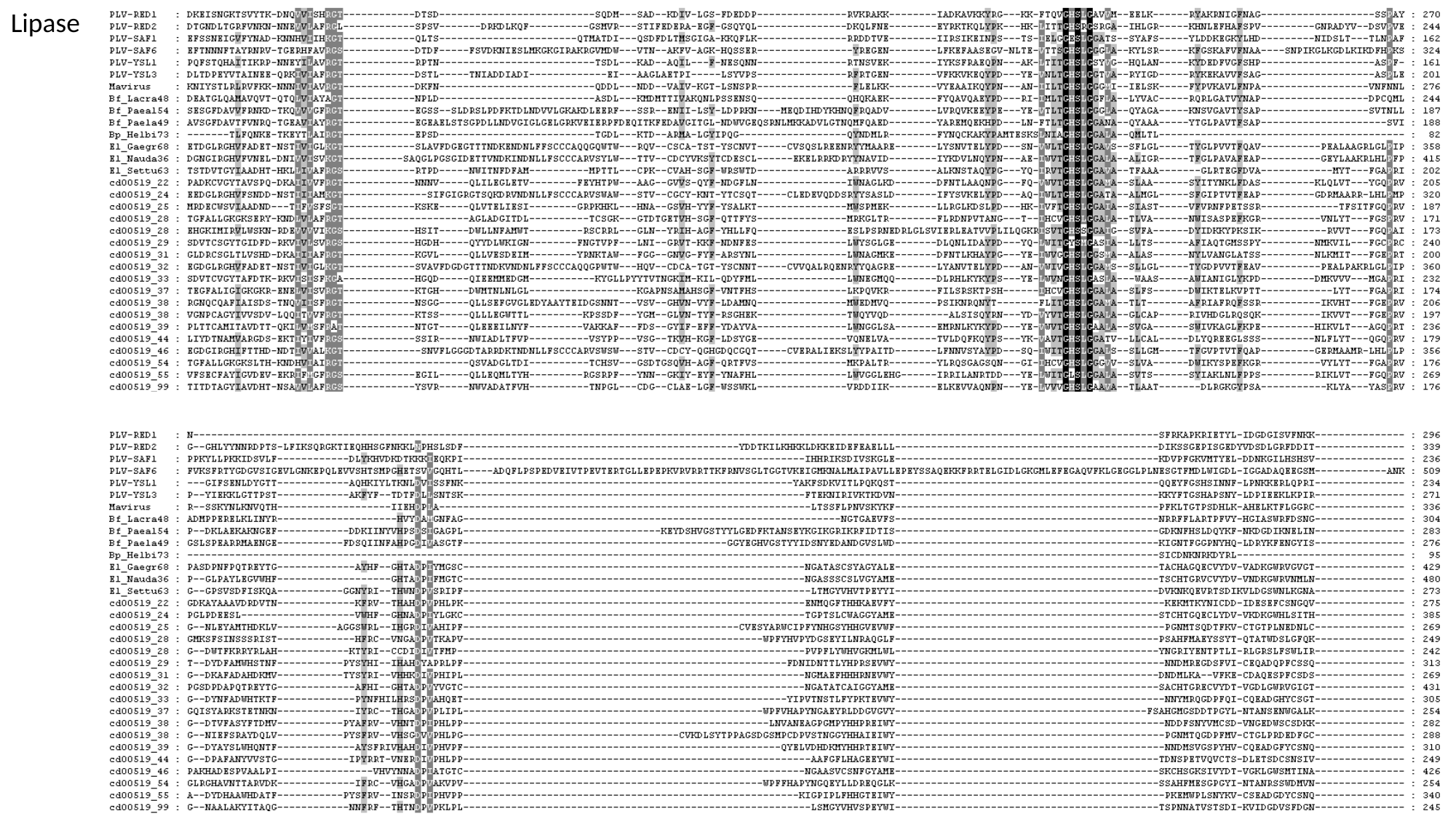

Lipase
